# Distinct bronchial microbiome precedes clinical diagnosis of lung cancer

**DOI:** 10.1186/s12943-022-01544-6

**Published:** 2022-03-07

**Authors:** Erin A. Marshall, Fernando S. L. Filho, Don D. Sin, Stephen Lam, Janice M. Leung, Wan L. Lam

**Affiliations:** 1 Department of Integrative Oncology, BC Cancer Research Institute, Vancouver, BC Canada; 2 Interdisciplinary Oncology Program, University of British Columbia, Vancouver, BC Canada; 3 Centre for Heart Lung Innovation, St Paul’s Hospital, Vancouver, BC Canada

**Keywords:** Lung cancer, Early detection, Microbiome, Risk prediction, Liquid biopsy

## Abstract

Resident microbial populations have been detected across solid tumors of diverse origins. Sequencing of the airway microbiota represents an opportunity for establishing a novel omics approach to early detection of lung cancer, as well as risk prediction of cancer development. We hypothesize that bacterial shifts in the pre-malignant lung may be detected in non-cancerous airway liquid biopsies collected during bronchoscopy. We analyzed the airway microbiome profile of near 400 patients: epithelial brushing samples from those with lung cancer, those who developed an incident cancer, and those who do not develop cancer after 10-year follow-up. Using linear discriminate analysis, we define and validate a microbial-based classifier that is able to predict incident cancer in patients before diagnosis with no clinical signs of cancer. Our results demonstrate the potential of using lung microbiome profiling as a method for early detection of lung cancer.

## Background

Forecasting the 10-15% of smokers who would develop lung cancer would improve survival rate [1, 2]. Smoking weakens the integrity of the bronchial epithelium, rendering the lungs more susceptible to resident microbial changes [3, 4]. Lung tumor and surround non-malignant tissues show differences in microbiome composition, and are both distinguishable from health lungs [4–8]. While lung microbiome profiles are explored as a marker for tumor presence, we see far greater potential in the clinical utility of detecting lung microbiome changes, as an indicator of imminent cancer development, in individuals at risk for cancer before any cancer diagnosis can be made, i.e. prior to clinical detection of tumors [9]. Here, we examined airway microbiomes of 400 individuals prior to cancer diagnosis, and compared those who do and do not subsequently develop cancer within a ~10-year follow-up period**.**

## Results

### Patient cohorts and microbiome profiling

**Fig. 1:**
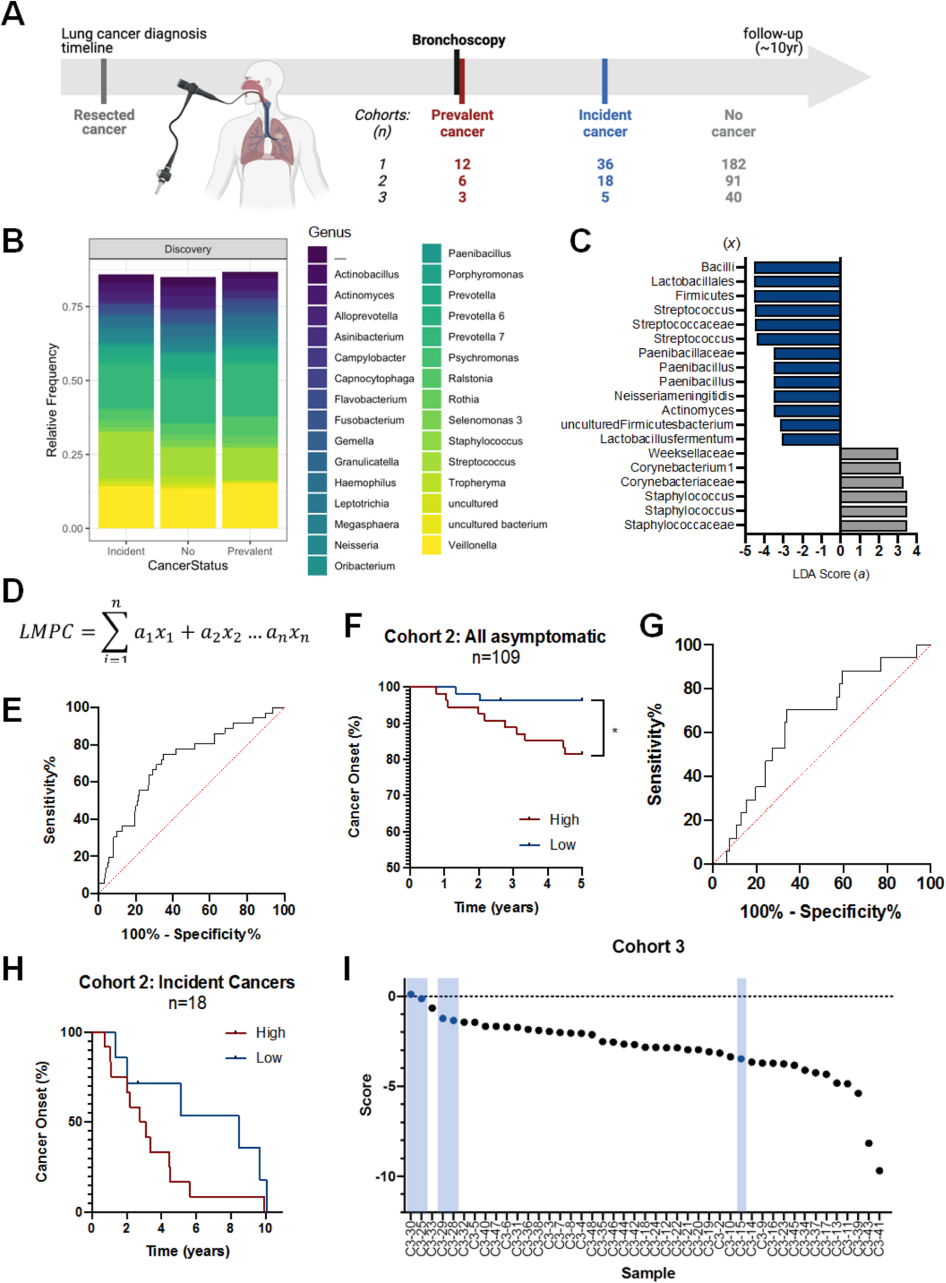
Microbiome-based LMPC classifier identifies cancer onset in independent cohort of patients at risk of lung cancer. **A** Timeline of bronchial sampling in Cohorts 1-3. **B** Relative abundance of taxa in the microbial communities Cohorts 1 is shown at genus level of classification. **C** Linear discriminant analysis (LDA) scores of taxonomic features included in the LMPC scoring model. A score magnitude of 4 was used as a cut-off for significant features, with a Kruskal-Wallis *p*-value of <0.05. Grey bars represent taxa that are higher in relative abundance in No Cancer participants, while blue bars represent taxa with higher relative abundance in Incident Cancer participants. **D** Formula used to construct LMPC score, where *a* represents the LDA score value and *x* represents the relative abundance of each taxa (described in Fig. 1C). **E** Receiver Operating Characteristic AUC differentiates Incident Cancer participants from No Cancer participants in Cohort 1 (*p*<0.0001, AUC: 0.7057, 95% CI: 0.6118 to 0.7997). **F** When the Incident Cancer (*n* = 18) and No Cancer participants (*n* = 91) in Cohort 2 were combined and stratified by risk score, those with high scores (red) had significantly earlier cancer diagnosis than those with low score (blue). **G** Within the Incident Cancer patient group, those with high scores demonstrated shorter time to cancer diagnosis than those with low scores. In both analysis, samples were separated into two groups based on the median score value. **H** Using a Receiver Operating Characteristic AUC as defined by the score, we are able to differentiate Incident Cancer participants from No Cancer participants in Cohort 2 (*p*=0.0498, AUC: 0.6503, 95% CI: 0.5167-0.7839). **I** In an independent cohort (Cohort 3), incident cancer participants comprised 4 of the top 5 largest score values. Incident cancer participants are shown in blue, while participants who did not have a diagnosed cancer with follow-up are indicated in black. Cancer onset was assessed using receiver operating curves and log-rank tests, where *p*<0.05 was considered significant

Bronchscopy-obtained tissue and liquid biopsies (bronchial brushing and washing) are clinically used to monitor high-risk patients. Bronchial brushing samples from bronchoscopy of 352 smokers were retrospectively obtained as part of the Lung Health Study at BC Cancer (BCC) with Research Ethics Board approval [10]. Seven patients had a previous diagnosis of lung cancer that was treated with surgery. Study participants were stratified based on their diagnosis at the time of sampling by bronchoscopy into No Cancer, Incident Cancer and Prevalent Cancer categories for correlative analyses with microbiome attributes (Fig. 1A). Incident Cancer refers to patients who at the time of bronchoscopy did not have cancer, but during follow-up developed incident lung cancer. The 345 samples were randomized and arbitrarily divided into two cohorts such that each category was matched for age, pack-year smoking history, and follow-up time between cohorts: two-thirds used for discovery, and one-third as validation (Cohort 1 *n* = 230, Cohort 2 *n* = 115; Table 1). These cohorts show no statistically-significant difference in age, pack-year smoking history, lung function, or follow-up time (all *p*>0.05; mean follow-up of 10.2 years).

**Table 1:** Clinical summary of Cohort 1 (*n*=230), 2 (*n*=115), and 3 (*n*=48). Cohorts 1 and 2 were sourced from BCC, and clinical characteristics are summarized for incident- (*n*=36, n=18), prevalent- (*n*=12, n=6), and no-cancer (*n*=182, *n*=91) participants. Cohort 3 was sourced from SPH/BCC and clinical characteristics are summarized for incident- (*n*=5), prevalent- (*n*=3), no-cancer (*n*=40) participants. Patients were randomly assisnged to Cohort 1 or 2 Values are displayed as mean values, unless otherwise specified

	Cohort 1			Cohort 2			Cohort 3		
	Incident	Prevalent	No cancer	Incident	Prevalent	No cancer	Incident	Prevalent	No cancer
**N**	**36**	**12**	**182**	**18**	**6**	**91**	**5**	**3**	**40**
**Age (mean)**	64.4	62.1	62.1	64.6	65.5	61.8	64.6	64.3	62.6
**Sex (% female)**	44.4	50	46.2	50	83.3	45.1	40	66.6	47.5
**Smoking status (mean %)**									
**Current**	50	25	45.6	55.6	33.3	47.3	20	33.3	47.5
**Ex**	50	75	54.4	44.4	66.7	52.7	60	0.0	52.5
**Never**	0	0	0	0	0	0	20	33.3	0
**Pack-years smoked (mean)**	51.1	48	47	55.3	47	44.8	50.7	37.8	48
**Lung function (mean)**									
**FEV1/FVC**	67.3	62	71.3	66	65.2	72.1	73.6	79	67
**FEV1**	2.5	2.1	2.6	2.3	1.8	2.6	2.3	2.1	2.4
**FEV1 % predicted**	78.5	71.4	84.3	85.2	68.6	85.2	88	83.3	77.6
**COPD status (mean %)**									
**No COPD**	36.1	33.3	57.1	38.9	33.3	63.7	80	100	45
**Mild**	19.4	8.3	18.1	16.7	0	17.6	0	0	5
**Moderate**	38.9	25	20.9	33.3	33.3	16.5	20	0	35
**Severe**	2.8	16.7	2.7	11.1	16.7	1.1	0	0	15
**Very Severe**	0	0	0	0	0	0	0	0	0
**Follow-up time (mean, yr)**									
**Total**	9.7	9.5	10	10.1	8.4	10.1	7.6	7	9.8
**To cancer**	4.2	N/A	N/A	4.3	N/A	N/A	1.9	N/A	N/A
**Cancer type**									
**LUAD**	42.4	88.9		38.9	66.7		100	100	
**LUSC**	12.1	11.1		16.7	33.3		0	0	
**NSCLC**	9.1	0		11.1	0		0	0	
**SCLC**	12.1	0		5.6	0		0	0	
**Other**	24.2	0		27.8	0		NA	NA	

Airway DNA samples were amplified and profiled at the hypervariable region V4 of the 16S rDNA [11] using a paired-end read chemistry (2x250bp) alongside negative control samples for amplification and sequencing. All samples yielded high-quality sequence data for delineating operational taxonomic units (OTUs, Naïve Bayes clustering) through clustering and alignment to the SILVA reference database (v132) [12, 13]. Reads were processed using the QIIME2® analysis platform (version 2019.1) [14], and forward reads were trimmed to 231 bases. Organisms identified to be dominant in control samples were excluded from analysis. Microbial community alpha diversity was evaluated (Shannon index, Pielou Evenness, number of OTUs, and Faith’s phylogenetic diversity), and no significant difference in diversity between Cohorts 1 and 2, or between groups within each cohort. Further, principal coordinate analysis of Bray Curtis Dissimilarity did not result in separation between participants from Cohorts 1 and 2 (PERMOVA, *p*>0.05; data not shown). Additional samples were obtained from a BC Cancer and St. Paul’s Hospital study to form a third cohort for the validation of the microbiome classifier for predicting incident cancers (Cohort 3, *n* = 48; described in Table 1).

### Relating airway microbiome to cancer status

At the Genus level, relative abundance measures were dominated by *Veillonella, Streptococcus, Prevotella,* and *Paenibacillus* in all three categories (182 No Cancer, 36 Incident Cancer and 12 Prevalent Cancer) (Fig. 1B). Taxonomic pattern in Cohort 1 discriminated the Incident Cancer Group from the No Cancer Group (i.e. no lung cancer at any point during follow-up) suggesting that shifts in taxonomic composition related to lung cancer may occur in airways of patients who develop incident lung cancer many months from the date of bronchoscopy. To determine which taxonomic identifiers discriminated patients who developed incident cancers from those who did not develop lung cancer, we used a combination of linear discriminate analysis and effect size modeling (LEfSe; Fig. 1C) [15]. LEfSe was chosen to determine features most likely differentiate groups because in additional to differences in relative abundance, this statistical model captures potential relevance by considering magnitude effect size in the LDA score. Beyond the relative abundance of *Bacilli*, the features with the strongest association with incident cancer status were concentrated in the Bacilli class, and the relative abundance of *Lactobacillales*, the *Streptococcus* genus and associated family, as well as the *Paenibacillus* genus and associated family.

### LMPC classifier predicting incident lung cancer in smokers

To determine whether the candidate features identified above could be used to build a classifier to predict incident cancer risk, we created a summative score based on the LDA scores and applied them to our cohorts (LMPC; Lung Microbiome Predictor of Cancer). A LDA-weighted combined score was created by multiplying the LDA score value (established in the discovery cohort above) by the relative frequency of the feature in each of the patient sample (Fig. 1D).

In the discovery cohort, the Area Under the Curve of the Receiver Operating Characteristic (AUC) was able to differentiate the Incident Cancer participants from the No Cancer participants (*p*<0.0001, AUC: 0.7057, 95% CI: 0.6118-0.7997; Fig. 1E). Using Cohort 2 as a validation cohort (18 Incident Cancer and 91 No Cancer), this classifier was able to predict cancer incidence. When stratified by the median score, patients with high scores had a significantly higher rates of incident cancer than those with low scores (*p*=0.01) (Fig. 1F). Further, when clinical features are incorporated to adjust for participant age, smoking history (pack-years smoked), and lung function (FEV1% predicted), the LMPC score can identify patients who will develop lung cancer (Cox proportional hazards model; log-rank test, *p*=0.004). Again, the AUC was able to differentiate the Incident Cancer from No Cancer participants (*p*=0.0498, AUC: 0.6503, 95% CI: 0.5167-0.7839) (Fig. 1G). Further, within the Incident Cancer subset of Cohort 2, patients with higher scores had a trend towards faster cancer incidence than those with low scores (log-rank test, *p*=0.05; adjusted for clinical co-variants as above) (Fig. 1H).

### Application of LMPC classifier to an independent cohort

A third cohort was processed and sequenced independently from Cohorts 1 and 2 (40 No Cancer, 5 Incident Cancer). After applying the scoring system to Cohort 3, 4/5 Incident Cancer participants had the higher risk scores, indicating that these individuals may be at the highest risk of developing cancer (Fig. 1I). Indeed, they developed lung cancer on average 16 months from the time of sampling, while the case with low score did not developed cancer until 53 months. The AUC readily differentiated Incident Cancer from No Cancer participants (*p*=0.0103, AUC: 0.8550, 95% CI: 0.6180-1.000).

## Discussion

Distinguishing the 10-15% of smokers who would develop lung cancer from those who will not presents a clinical challenge. While the consensus of specific taxonomic classifiers associated with lung cancer remains contentious, it is clear that the microbiome is involved in human biological processes, and plays a functional role in cancer pathogenesis. However, the timing of these microbial community alterations with respect to cancer onset remains unknown and has tremendous clinical implications in advancing early detection of lung cancer. Here, we showed that there are significant detectable changes in the lung microbiome in participants who, at the time of bronchoscopy, did not have cancer but who subsequently developed lung cancer during follow-up (i.e. the Incident Cancer patients). These data thus propose a microbial-DNA-based classifier to predict incident cancer cases in our cohort of current and former smokers.

We identified taxonomic features (changes in Cohort 1) that yielded the largest LDA score and developed a classifier, which was able to distinguish cancer status. The classifier model was validated in Cohort 2, demonstrating its ability to distinguish Incident Cancer from No Cancer and thus enabling prediction of incident cancer in smokers who are otherwise asymptomatic (Fig. 1D). When stratified by the LMPC classifier, participants with a high score have a significantly shorter time to cancer onset than those with a low score in a combined group of No-Cancer and Incident Cancer participants. Of note, the absolute risk in this cohort is consistent with cancer onset rates in “high-risk” individuals, suggesting that these findings may be applicable to a broader at-risk community. Within the Incident Cancer group (in Cohort 2) those samples with high scores (separated by median) had earlier cancer onset than those with low scores (Fig. 1F).

When this same scoring was applied to a third cohort that was sequenced independently, 4/5 Incident Cancer participants were identified to have the highest LMPC scores and developed cancer in a mean time of 1.29 years from the time of sampling (Fig. 1H). Upon further examination, the fifth Incident Cancer sample (C3-15) with the lower score was found to have a much longer time-to-cancer diagnosis of 4.43 years following the airway sample collection. Since this proposed classifier is able to distinguish participants who will develop cancer over time from those who will not, it is reasonable to expect that this score is more predictive the closer the sample procurement is to cancer diagnosis. The behavior of this patient is consistent with this expectation, indicating that this risk score model may be most applicable closer to cancer onset.

Our results demonstrate that specific changes in airway liquid biopsy (brushings) samples, obtained during clinical examination by bronchoscopy, in fact, occur before clinical cancer diagnosis. Lung microbiome shifts can serve as a marker in advance of the clinical phenotypes detectable by low-dose CT scans. We have defined a microbiome-based classifier that can predict incident cancer with follow-up from patients having no clinical signs of cancer. As lung-resident microbiomes are associated with smoking status, and this study describes cohorts of ever-smokers and former smokers, future work should examine if this signature can be applied to assess cancer onset in individuals without a history of smoking history. Our findings provide strong rationale for developing microbiome-based liquid biopsy technology to prioritize at-risk individuals for clinical attention.

## Data Availability

All sequencing data generated in this study have been deposited in the Sequence Read Archive (SRA) of the National Center for Biotechnology Information (NCBI).

## Abbreviations

AUC: Area under the curve
BCC: BC Cancer
LDA: Linear discriminant analysis
LDCT: Low-dose computed tomography
LEfSe: Linear discriminant analysis effect size
LHS: Lung health study
LUAD: Lung adenocarcinoma
LUSC: Lung squamous cell carcinoma
FEV: Forced expiratory volume
FEV1: Forced expiratory volume in 1 second
FVC: Forced Vital Capacity
NM: Non-malignant
NSCLC: Non-small cell lung cancer
OTU: Operational taxonomic unit
SCLC: Small cell lung cancer
SPH: St Paul’s Hospital
